# Transcriptional response of soybean to thiamethoxam seed treatment in the presence and absence of drought stress

**DOI:** 10.1186/1471-2164-15-1055

**Published:** 2014-12-03

**Authors:** Mitchell D Stamm, Laramy S Enders, Teresa J Donze-Reiner, Frederick P Baxendale, Blair D Siegfried, Tiffany M Heng-Moss

**Affiliations:** Department of Entomology, University of Nebraska, 103 Entomology Hall, Lincoln, NE 68583 USA

**Keywords:** Neonicotinoid, Next-generation sequencing, Stress shield

## Abstract

**Background:**

Neonicotinoid insecticides are widely known for their broad-spectrum control of arthropod pests. Recently, their effects on plant physiological mechanisms have been characterized as producing a stress shield, which is predicted to enhance tolerance to adverse conditions. Here we investigate the molecular underpinnings of the stress shield concept using the neonicotinoid thiamethoxam in two separate experiments that compare gene expression. We hypothesized that the application of a thiamethoxam seed treatment to soybean would alter the expression of genes involved in plant defensive pathways and general stress response in later vegetative growth. First, we used next-generation sequencing to examine the broad scale transcriptional effects of the thiamethoxam seed treatment at three vegetative stages in soybean. Second, we selected ten target genes associated with plant defense pathways in soybean and examined the interactive effects of thiamethoxam seed treatment and drought stress on expression using qRT-PCR.

**Results:**

Direct comparison of thiamethoxam-treated and untreated soybeans revealed minor transcriptional differences. However, when examined across vegetative stages, the thiamethoxam seed treatment induced substantial transcriptional changes that were not observed in untreated plants. Genes associated with photosynthesis, carbohydrate and lipid metabolism, development of the cell wall and membrane organization were uniquely upregulated between vegetative stages in thiamethoxam-treated plants. In addition, several genes associated with phytohormone and oxidative stress responses were downregulated between vegetative stages. When we examined the expression of a subset of ten genes associated with plant defense and stress response, the application of thiamethoxam was found to interact with drought stress by enhancing or repressing expression. In drought stressed plants, thiamethoxam induced (upregulated) expression of a thiamine biosynthetic enzyme (THIZ2) and gibberellin regulated protein (GRP), but repressed (downregulated) the expression of an apetala 2 (GmDREB2A;2), lipoxygenase (LIP), and SAM dependent carboxyl methyltransferase (SAM).

**Conclusions:**

We found evidence that a thiamethoxam seed treatment alters the expression soybean genes related to plant defense and stress response both in the presence and absence of drought stress. Consistent with the thiamethoxam stress shield concept, several genes associated with phytohormones showed enhanced expression in drought stressed plants.

**Electronic supplementary material:**

The online version of this article (doi:10.1186/1471-2164-15-1055) contains supplementary material, which is available to authorized users.

## Background

Neonicotinoid insecticides have become a fast-growing class of insecticides since imidacloprid first became commercially available in 1991 [1]. The effectiveness of these products has been well-documented for control of a broad-spectrum of sucking and chewing insect pests on major crops [2]. In recent years, seed treatments containing neonicotinoid insecticides, including imidacloprid, clothianidin, and thiamethoxam, have become a popular management option for growers. When applied to seeds, neonicotinoids distribute systemically throughout the plant and provide protection against early-season insect pests feeding on leaf tissue or plant sap [2].

Recently, it has been suggested that neonicotinoid insecticides can affect physiological mechanisms involved with plant health [3]. Research in row, vegetable, and fruit crops suggests plant growth is enhanced with a foliar or soil drench application of imidacloprid [3]. In cotton, a foliar application of imidacloprid was shown to increase peroxidase enzyme activity, phenol content, plant height, boll numbers and overall yield [4]. In addition, imidacloprid and clothianidin induced the expression of genes associated with salicylic acid-mediated defense when applied as a foliar and soil treatment in *Arabidopsis thaliana* [3, 5]. This pathway is commonly associated with abiotic stress response [6, 7], and has led to the concept of a protective stress shield induced by neonicotinoids [3]. This unique mode of action is predicted to help plants temper the effects of abiotic and biotic stress [3]. However, foliar lesions and peroxidative damage have also been documented in plants, including soybean, when several different neonicotinoids were applied hydroponically [8]. These data suggest that neonicotinoids may cause unanticipated oxidative stress that could negatively affect aspects of plant growth and development.

Thiamethoxam is widely used in cropping systems [2] and a growing number of studies have demonstrated beneficial effects on plant growth and development [9–14]. Studies have begun to explore the physiological and molecular mechanisms that contribute to enhanced plant vigor associated with its application [10–13], but a comprehensive analysis of the transcriptional changes in response to thiamethoxam is lacking. In addition, it is unclear whether thiamethoxam alters plant responses to environmental stress. For example, phytohormones associated with plant defense pathways, shown to respond to drought stress in late vegetative and early reproductive stage soybeans [15], may also be affected by the thiamethoxam seed treatment. In soybean, an application of thiamethoxam improved germination under drought stress [9]. However, the interactive effects of seed treatment and drought stress have yet to be investigated at the molecular level in soybean. This may provide important insight into the potential for these seed treatments to protect plants from important environmental stressors.

Advances in next-generation sequencing (RNA-Seq) technologies combined with the sequencing of the soybean genome [16] make it possible to investigate the complex interactions between common agricultural practices (e.g., seed treatments) and environmental stress at the transcriptional level. Two gene expression experiments were designed to advance our understanding of the interactions among seed treatments, plant defenses, and drought stress. The first objective of this study was to characterize the effects of a thiamethoxam seed treatment on gene expression in soybean in the absence of stress. Gene expression levels in thiamethoxam-treated and untreated plants were compared at three different vegetative stages using RNA-Seq. The second objective was to investigate the interactive effects of seed treatment and drought stress on a suite of genes associated with established plant defensive pathways in soybean. We used qRT-PCR to compare gene expression of thiamethoxam-treated and untreated soybeans exposed to drought stress and unstressed (control) conditions of ten selected genes involved in plant defense pathways and general stress response. We hypothesized that thiamethoxam would alter the expression of genes involved in plant defense pathways and general stress response. In addition, we hypothesized that changes in gene expression caused by the thiamethoxam seed treatment would be more pronounced when drought stress was applied.

## Results

### Experiment I: transcriptional response to thiamethoxam seed treatment

Across all vegetative stages the average number of reads that aligned to the soybean genome was 27.5 (92.32%) and 26.5 million (91.87%) for the thiamethoxam-treated and untreated control samples, respectively. Specifically, the total mean number of reads for the thiamethoxam-treated VC, V2, and V4 samples were 29.5, 28.5, and 31.0 million, respectively. For the untreated control VC, V2, and V4 samples, the total mean number of reads were 29.0, 26.0, and 32.0 million, respectively (Table 1).

**Table 1:** **Statistics of total reads and alignment generated from the Bowtie 2**

Harvest stage	Treatment	Mean total reads	Mean total aligned	Mean total aligned (%)	Mean total failed (%)
VC	Thiamethoxam	29,482,102.67	27,471,240.33	93.19	6.81
VC	Untreated	28,802,062.33	26,775,525.67	92.89	7.11
V2	Thiamethoxam	28,473,131.00	25,602,095.67	89.77	10.23
V2	Untreated	25,731,660.33	22,573,929.67	87.96	12.04
V4	Thiamethoxam	31,245,900.33	29,373,971.00	94.00	6.00
V4	Untreated	32,196,256.33	30,505,024.00	94.75	5.25

The direct effects of thiamethoxam on gene expression were minor at the V2 and V4 stages and there were no significant differences in gene expression between the thiamethoxam-treated and untreated control VC soybeans (Table 2). In V2 soybeans, four differentially expressed (DE) genes were downregulated, including two aquaporins (Table 2); however, the effect of thiamethoxam was minimal since fold changes were small (-1.8 – 2.8). In V4 soybeans, two genes were downregulated, including a Myb transcription factor (*Glyma09g04370*; Table 2). A total of four genes were upregulated in the thiamethoxam-treated plants (Table 2). In V2 soybeans, a Rare lipoprotein A-like (RlpA) double-psi beta-barrel and undescribed gene were upregulated by 2.0 and 1.8-fold, respectively. In V4 soybeans, a terpene synthase and mediator complex subunit 28 was upregulated by 5.8 and 3.5-fold in the thiamethoxam-treated plants.

**Table 2:** **Differentially expressed genes between treatments (thiamethoxam-treated relative to untreated) at the V2 and V4 vegetative stages (** ***p*** **adj ≤ 0.05, fold change ≥ 2.0)**

Gene ID	Gene name	Fold change
**V2**		
*Glyma12g06730*	Rare lipoprotein A-like (RlpA) double-psi beta-barrel	2.0
*Glyma02g31120*	NA	1.8
*Glyma02g43740*	Oligopeptide transporter-related	-1.8
*Glyma05g14800*	Plastocyanin-like domain	-2.2
*Glyma18g42630*	Aquaporin	-2.7
*Glyma02g08120*	Aquaporin	-2.8
**V4**		
*Glyma13g32380*	Terpene synthase	5.8
*Glyma08g13870*	Mediator complex subunit 28	3.5
*Glyma09g04370*	Myb transcription factor	-4.8
*Glyma03g31410*	NA	-6.9

Differential gene expression in the VC comparison was not detected.

**Figure 1:**
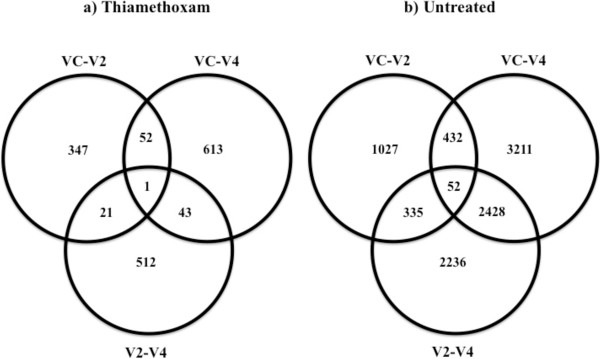
**Venn diagrams displaying overlapping DE genes (up- and downregulated combined) that are unique to the seed treatment. a)** thiamethoxam-treated and **b)** untreated soybeans found in three vegetative stage comparisons (VC-V2, VC-V4, and V2-V4).

Further characterization of thiamethoxam-induced changes in gene expression included comparisons between each respective vegetative stage (VC-V2, VC-V4, and V2-V4) within each experimental treatment. This resulted in a set of three vegetative comparisons for both thiamethoxam-treated plants and untreated controls (6 total comparisons). Overall, fewer DE genes (up- and downregulated) were identified between vegetative stages in the thiamethoxam-treated plants compared to untreated controls (Table 3). This pattern was also observed when we focused on the DE genes identified in vegetative stage comparisons that were unique to thiamethoxam-treated plants (i.e., no DE in untreated controls) or unique to the untreated controls (i.e., no DE in thiamethoxam-treated plants; Figure 1). In thiamethoxam-treated plants, only one gene, an aspartic protease (*Glyma11g03500*), was DE across all vegetative stage comparisons. In contrast, 52 genes were identified as being DE among all vegetative stage comparisons in the untreated controls (Figure 1). These results suggest that majority of the transcriptional changes in both treatments are primarily stage-specific (Figure 1).

**Table 3:** **Total number of DE genes between vegetative stages for thiamethoxam-treated and untreated control soybeans (** ***p*** **adj ≤ 0.05)**

Growth stage comparison		Number of genes		
	Treatment	Upregulated	Downregulated	Total
VC-V2	Thiamethoxam	4887	4567	9454
VC-V2	Untreated	6227	6211	12438
V2-V4	Thiamethoxam	3543	5193	8736
V2-V4	Untreated	5558	6462	12020
VC-V4	Thiamethoxam	4708	5446	10154
VC-V4	Untreated	7982	8047	16029

### Characterization of biological functions associated with transcriptional changes

Enrichment analysis of DE genes in thiamethoxam-treated plants that were upregulated in all three vegetative stage comparisons were associated with photosynthesis, carbohydrate synthesis, cell wall and membrane development, membrane organization, and lipid metabolism (Table 4). These GO terms were not enriched in the corresponding vegetative stage comparisons of the untreated control plants (Additional file 1: Table S1 and Additional file 2: Table S2).

**Table 4:** **Enrichment of GO Terms (FDR < 0.10) of DE upregulated genes in the thiamethoxam VC-V2, VC-V4, and V2-V2 comparisons**

a. Thiamethoxam VC-V2				
GO ID	GO description	Gene ID	Gene name	Fold change
*Cellular components and photosynthetic-related processes*				
GO:0009534	Chloroplast thylakoid	*Glyma04g22110*	Photosystem I protein	16.3
GO:0009579	Thylakoid	*Glyma01g07130*	Cytochrome b	3.0
GO:0015979	Photosynthesis	*Glyma12g36140*	Cytochrome b559	2.8
GO:0022900	Oxidation-reduction process	*Glyma15g12130*	Cytochrome b	2.8
GO:0022900	Electron transport chain			
GO:0031976	Plastid thylakoid			
GO:0034357	Photosynthetic membrane			
GO:0042651	Thylakoid membrane			
GO:0044436	Thylakoid part			
**b. Thiamethoxam VC-V4**				
**GO ID**	**GO description**	**Gene ID**	**Gene name**	**Fold change**
*Biological processes related to carbohydrate synthesis*				
GO:0005975	Carbohydrate metabolic process	*Glyma19g41100*	Nucleotidyltransferase	6.1
GO:0006073	Cellular glucan metabolic process	*Glyma06g13480*	Glycosyltransferases	5.0
GO:0044042	Glucan metabolic process	*Glyma15g43040*	Cellulose synthase	3.3
GO:0044262	Cellular carbohydrate metabolic process	*Glyma15g15820*	Galactosyltransferase	3.2
GO:0044264	Cellular polysaccharide metabolic process	*Glyma04g07220*	Cellulose synthase	2.8
		*Glyma02g47460*	Nucleotidyltransferase	2.8
		*Glyma06g03340*	Nucleotidyltransferase	2.3
		*Glyma12g36570*	Cellulose synthase	2.0
*Biological processes related to cell wall development*				
GO:0005618	Cell wall	*Glyma06g05290*	Glycosyl hydrolase family 10	6.2
GO:0010383	Cell wall polysaccharide metabolic process	*Glyma04g05220*	Glycosyl hydrolase family 10	5.8
GO:0044036	Cell wall macromolecule metabolic process	*Glyma14g02070*	Glycerophosphoryl diester phosphodiesterase	2.1
GO:0071554	Cell wall organization or biogenesis			
**c. Thiamethoxam V2-V4**				
**GO ID**	**GO description**	**Gene ID**	**Gene name**	**Fold change**
*Cellular components related to cell membrane*				
GO:0012505	Endomembrane system	*Glyma11g01230*	Leucine rich repeat/protein tyrosine kinase	3.0
GO:0016021	Integral to membrane	*Glyma09g28190*	Cellulose synthase	2.4
GO:0031224	Intrinsic to membrane	*Glyma08g34790*	Leucine rich repeat/protein tyrosine kinase	2.1
GO:0044425	Membrane part	*Glyma20g05530*	HMG-CoA	2.1
*Biological processes related membrane organization and lipid metabolism*				
GO:0004630	Phospholipase D activity	*Glyma07g01310*	Phospholipase D	3.2
GO:0006629	Lipid metabolic process	*Glyma07g11580*	Methyltransferase	2.8
GO:0042439	Ethanolamine-containing compound metabolic process	*Glyma07g03490*	Phospholipase D	2.6
GO:0046470	Phosphatidylcholine metabolic process	*Glyma08g22600*	Phospholipase D	2.3
		*Glyma11g08640*	Phospholipase D	2.2

These DE genes were present only in the three thiamethoxam-treated comparisons. Condensed list contains responsive genes possibly associated with the thiamethoxam seed treatment. The genes listed were in common among the GO IDs. Fold change is relative to soybean development (e.g., the earlier vegetative stage).

In contrast, there was no significant enrichment of biological pathways found among the downregulated genes unique to thiamethoxam-treated plants. This was likely due to the low overall number of DE genes between the respective vegetative stage comparisons of the thiamethoxam-treated plants. However, several genes unique to the thiamethoxam-treated comparisons were associated with phytohormones and oxidative stress, including genes associated with jasmonic acid (JA) and gibberellin (GA) pathways, two phytohormone related pathways involved in plant defenses (Table 5). Additionally, several oxidative enzymes were downregulated, including peroxidases and cytochrome P450s (Table 5).

**Table 5:** **DE downregulated genes in the thiamethoxam VC-V2, VC-V4, and V2-V4 comparisons**

Gene ID	Gene name	Fold change
**VC-V2**		
*Glyma15g05580*	Cytochrome P450	-2.6
*Glyma03g29790*	Cytochrome P450	-25.8
*Glyma13g03790*	Lipoxygenase (JA-associated)	-41.7
*Glyma18g44320*	Peroxidase	-72.7
*Glyma13g20170*	Peroxidase	-301.6
**VC-V4**		
*Glyma05g28610*	Chalcone and stilbene synthases (JA-associated)	-3.3
*Glyma13g24200*	Cytochrome P450	-5.4
*Glyma03g29790*	Cytochrome P450	-15.4
*Glyma19g42940*	Cytochrome P450	-17.8
*Glyma05g26080*	2OG-Fe(II) oxygenase (JA- and GA-associated)	-24.0
*Glyma13g20170*	Peroxidase	-173.5
**V2-V4**		
*Glyma04g40000*	RlpA-like double-psi beta-barrel (GA-associated)	-2.5
*Glyma19g01470*	cytochrome P450	-2.6
*Glyma15g17620*	Peroxidase	-6.3
*Glyma08g43890*	Peroxidase/oxygenase (JA-associated)	-11.6

The genes presented were associated with phytohormones and oxidative stress response and unique to the thiamethoxam treatment.

### qRT-PCR Validation of RNA-Seq Results

Transcriptional changes measured between each soybean vegetative stage using RNA-Seq were verified with qRT-PCR. We selected four target genes from the thiamethoxam-treated and untreated plants (Additional file 3: Table S3). The overall correlation between fold changes estimated using RNA-Seq and qRT-PCR across vegetative stages (VC-V2, V2-V4, VC-V4) for each gene was 0.82 (Additional file 3: Table S3), indicating that transcriptional changes among growth stages in the selected genes were in agreement. A statistical comparison of qRT-PCR expression values using ANOVA and analysis of RNA-Seq data using DESeq also demonstrated that patterns of significant differential gene expression between vegetative stages were consistent between the two methods. Overall, 7/12 and 6/12 comparisons across all genes were in statistical agreement for thiamethoxam-treated and untreated plants, respectively (Additional file 3: Table S3).

### Experiment II: impact of drought stress on gene expression

#### Effects of drought stress on soybean growth

Drought stress had several negative effects on the physiological parameters measured in soybean. First, the effects of drought stress were observed in delayed vegetative growth of both thiamethoxam-treated and untreated plants relative to unstressed plants. Drought stressed plants did not develop past the V2 stage in contrast to unstressed plants that reached the V4 stage (13d), indicating a reduction in developmental rate. Second, drought stress caused a significant reduction in plant biomass. When averaged across all thiamethoxam-treated and untreated control plants, drought stressed plants showed a 1.4, 3.5, and 6.1-fold reduction in biomass compared to unstressed plants at 7, 10, and 13d, respectively (Table 6). Third, drought stress had a negative effect on plant height. Drought stressed thiamethoxam-treated and untreated control plants were on average 0.6, 5.0, and 10.4 cm shorter than unstressed plants at 7, 10, and 13d, respectively (Table 6). Taken together these results demonstrate that drought stress had a significant impact on soybean physiology and that the intensity of stress progressed over the three sampling dates both in thiamethoxam-treated and untreated control plants. However, we did not find that the thiamethoxam treatment enhanced overall plant vigor or response to drought stress. There were no significant differences in shoot height and plant biomass between thiamethoxam-treated and untreated control plants in the presence and absence of drought stress (Table 6).

**Table 6:** **The effects of drought stress and seed treatment on plant biomass (wet – dry weight) and shoot height (± SEM) at the three time points (7, 10, and 13d)**

		7d		10d		13d	
		Plant	Shoot	Plant	Shoot	Plant	Shoot
Treatment	Stress type	Biomass (g) ^a^	Height (cm)	Biomass (g)	Height (cm)	Biomass (g)	Height (cm)
Thiamethoxam	No stress	4.2 ± 0.1 a	11.1 ± 0.3 ab	6.3 ± 0.4 a	15.8 ± 0.4 a	9.3 ± 0.7 a	20.8 ± 0.4 a
Thiamethoxam	Drought stress	2.9 ± 0.1 b	10.7 ± 0.2 b	2.0 ± 0.1 b	11.3 ± 0.3 b	2.0 ± 0.1 b	11.9 ± 0.3 b
Untreated	No stress	4.5 ± 0.2 a	11.9 ± 0.2 a	6.8 ± 0.2 a	16.3 ± 0.2 a	10.7 ± 0.5 a	22.7 ± 0.3 c
Untreated	Drought stress	3.2 ± 0.2 b	11.2 ± 0.2 ab	1.8 ± 0.1 b	10.9 ± 0.3 b	1.4 ± 0.1 b	10.8 ± 0.2 b

^a^Treatment means within the same column followed by the same letter indicate no significant differences (*P* ≤ 0.05), LSD test.

### Interactive effects of thiamethoxam and drought stress on gene expression

Drought stress significantly altered gene expression of the ten target genes in at least one time point. A significant seed treatment and drought stress interaction was identified in the expression of several target genes. (Tables 7 and 8). Specifically, the effect of drought stress on gene expression varied between thiamethoxam-treated and untreated control plants at 7d (GmDREB2A;2, LIP, and SAM) and 13d (THIZ2, GRP, and GmDREB2A;2), as indicated by a significant seed treatment × drought stress interaction (Table 7). We found patterns consistent with increased expression in treated plants under stress and also decreased expression in treated plants under stress, which demonstrate interactive effects between thiamethoxam and drought stress.

**Table 7:** **Summaries of ANOVA with** ***P-*** **values for the overall effects of the stress (drought stress and no stress), seed treatment (thiamethoxam-treated and untreated control) and the two-way interaction of stress and seed treatment on target genes at the three time points (7, 10 and, 13d)**

	Effect of stress			Effect of seed treatment			Stress x seed treatment effect		
	7d	10d ^a^	13d	7d	10d	13d	7d	10d	13d
Target gene	***P-***value	***P-***value	***P-***value	***P-***value	***P-***value	***P-***value	***P-***value	***P-***value	***P-***value
GmDREB2A;2	**0.0195**	0.2119	0.1542	0.0777	0.3369	0.5096	**0.0112**	0.1430	**0.0059**
AP	0.9403	**<0.0001**	**0.0345**	0.7630	0.2951	0.7959	0.0640	0.3125	0.4484
CAR	**<0.0001**	**<0.0001**	**<0.0001**	**0.0101**	0.2775	0.2007	0.0775	0.2140	0.1037
P450	**0.0382**	**0.0007**	**0.0007**	**0.0238**	0.4635	0.1739	0.0554	0.6595	0.1413
GRP	**0.0058**	**0.0004**	**<.0001**	0.2914	0.2094	0.1673	0.6708	0.8425	**0.0126**
LIP	**0.0042**	0.6538	0.0729	**0.0012**	0.1806	**0.0488**	**0.0003**	0.5632	0.4947
SAM	**0.0348**	**0.0011**	**0.0027**	0.4068	0.2750	0.9872	**0.0048**	0.8995	0.3545
THIZ1	**0.0002**	**<.0001**	**<.0001**	0.5960	0.3248	0.8535	0.5197	0.6831	0.4601
THIZ2	**0.0130**	**<.0001**	**0.0120**	0.6869	0.5985	0.2061	0.4427	0.0649	**0.0054**
WRKY51	**<.0001**	**0.0002**	**<.0001**	0.3549	0.6054	0.2845	0.3686	0.9049	0.6846

Bold *P*-value indicates a significant stress, treatment or treatment and stress effect (*P* ≤ 0.05), LSD test.

**Table 8:** **Fold changes for the ten target genes at the three time points (7, 10, and 13d) in response to stress (drought stress [S] and no stress [NS]) and seed treatment (thiamethoxam [Thx]-treated and untreated control [Unt])**

	Stress and seed treatment comparison (Fold Change ± SEM)			
Target gene	Thx (S) /	Unt (S) /	Thx (S) /	Thx (NS) /
(Time point)	Thx (NS)	Unt (NS)	Unt (S)	Unt (NS)
**GmDREB2A;2**				
(7d)	-1.3 ± 0.9	9.7 ± 3.3	-7.4 ± 1.4	1.7 ± 0.7
(10d)	-1.1 ± 1.3	2.7 ± 0.9	-1.2 ± 1.3	2.4 ± 0.8
(13d)	-1.7 ± 0.3	4.1 ± 1.7	-3.4 ± 0.3	2.1 ± 0.4
**AP**				
(7d)	-1.9 ± 1.6	2.1 ± 0.6	-2.2 ± 1.2	1.7 ± 0.4
(10d)	-78.8 ± 51.8	-30.9 ± 21.8	-1.1 ± 1.3	2.3 ± 0.7
(13d)	-2.5 ± 1.2	-4.7 ± 0.1	2.0 ± 1.4	1.0 ± 2.3
**CAR**				
(7d)	2.4 ± 0.2	2.8 ± 0.3	-1.5 ± 0.2	-1.3 ± 0.1
(10d)	2.9 ± 0.3	2.5 ± 0.1	1.2 ± 0.1	-1.0 ± 0.0
(13d)	2.8 ± 0.3	3.8 ± 0.2	-1.2 ± 0.1	1.1 ± 0.8
**P450**				
(7d)	2.5 ± 0.5	7.7 ± 2.8	-13.5 ± 3.3	-4.3 ± 3.4
(10d)	-3.8 ± 0.2	-2.8 ± 0.7	1.0 ± 0.7	1.4 ± 1.1
(13d)	-3.9 ± 0.3	-12.8 ± 0.8	1.1 ± 0.8	-3.1 ± 1.8
**GRP**				
(7d)	-1.2 ± 0.0	-1.1 ± 0.7	1.0 ± 0.0	1.1 ± 0.0
(10d)	-1.4 ± 0.0	-1.4 ± 0.1	1.1 ± 0.0	1.1 ± 0.0
(13d)	-1.6 ± 0.1	-2.6 ± 0.0	1.5 ± 0.1	-1.1 ± 0.8
**LIP**				
(7d)	-6.0 ± 1.8	1.5 ± 0.2	-6.9 ± 2.1	1.3 ± 0.9
(10d)	1.0 ± 1.0	-1.2 ± 0.9	-1.2 ± 1.0	-1.4 ± 1.1
(13d)	-1.5 ± 0.3	-2.0 ± 0.7	2.2 ± 0.5	1.6 ± 1.1
**SAM**				
(7d)	-4.2 ± 1.2	1.3 ± 0.1	-2.7 ± 0.8	2.1 ± 0.5
(10d)	-3.4 ± 0.7	-2.9 ± 0.4	-1.3 ± 0.3	-1.1 ± 1.1
(13d)	-2.6 ± 1.6	-4.6 ± 0.5	1.5 ± 1.2	-1.2 ± 1.2
**THIZ1**				
(7d)	-1.1 ± 0.0	-1.1 ± 0.0	-1.0 ± 0.7	1.0 ± 0.0
(10d)	-1.2 ± 0.0	-1.1 ± 0.0	1.0 ± 0.7	1.0 ± 0.0
(13d)	-1.2 ± 0.0	-1.3 ± 0.0	1.0 ± 0.0	-1.0 ± 0.7
**THIZ2**				
(7d)	-3.2 ± 1.5	-2.1 ± 0.6	-1.4 ± 1.3	1.1 ± 1.0
(10d)	-2.4 ± 0.3	-4.3 ± 0.4	1.3 ± 0.2	-1.4 ± 0.4
(13d)	1.2 ± 1.0	-6.6 ± 1.6	1.9 ± 0.5	-4.2 ± 1.0
**WRKY51**				
(7d)	-1.4 ± 0.1	-1.3 ± 0.0	-1.1 ± 0.0	-1.0 ± 0.7
(10d)	-1.6 ± 0.0	-1.6 ± 0.1	-1.1 ± 0.0	-1.0 ± 0.8
(13d)	-1.7 ± 0.0	-1.8 ± 0.1	1.1 ± 0.0	1.0 ± 0.7

The expression of GmDREB2A;2, a drought-responsive transcription factor [17], was affected by thiamethoxam. This gene was also shown to be stress-responsive and had significant interactions with the seed treatment at 7 and 13d (*F* = 10.76; df = 1, 8; *P* = 0.0112 and *F* = 13.83; df = 1, 8; *P* = 0.0059, respectively; Table 7). In thiamethoxam-treated plants at 7d, GmDREB2A;2 expression was repressed by -1.3-fold under drought stress compared to untreated control plants, which showed a significant 9.7 fold upregulation under stress (*t* = 4.38; df = 8; *P* = 0.0101; Table 8). This similar expression pattern was also observed at 13d (*t* = 3.75; df = 8; *P* = 0.0563) with GmDREB2A;2 being repressed in thiamethoxam-treated plants under drought stress by -1.7-fold compared to the untreated controls, which showed a significant 4.1-fold upregulation (Table 8).

**Figure 2:**
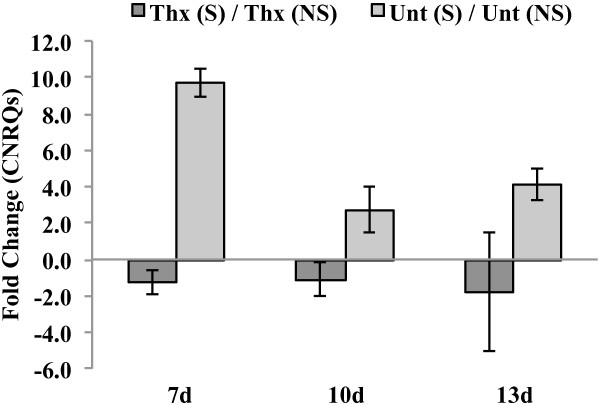
**Expression pattern of GmDREB2A;2 (apetala 2).** There was significant seed treatment x drought stress interaction under no stress (NS) and drought stress (S) at 7 and 13d. Under drought stress, the expression of GmDREB2A;2 was downregulated in thiamethoxam-treated (Thx) plants and upregulated in the untreated control (Unt).

Thiamethoxam also affected the expression of THIZ2, GRP, LIP, and SAM, which are associated with phytohormones. THIZ2, a thiamine biosynthetic enzyme, had a significant seed treatment × drought stress interaction at 13d (*F* = 14.25; df = 1, 8; *P* = 0.0054; Table 7), which was further investigated by comparing gene expression between drought-stressed and unstressed plants using Fisher’s LSD test (Table 8). Specifically, THIZ2 expression was upregulated under drought stress in thiamethoxam-treated plants, but significantly downregulated in the untreated controls at 13d (*t* = 4.95; df = 8; *P* = 0.0049; Table 7). GRP, a GA regulated protein, also had a significant seed treatment x drought stress interaction at 13d (*F* = 10.25; df = 1, 8; *P* = 0.0126; Table 7). This gene was downregulated in response to drought stress in both the thiamethoxam-treated and untreated control plants. However, the magnitude of the effect of drought stress on GRP expression in thiamethoxam-treated plants was less than observed in untreated controls (-1.6-fold compared to -2.6 fold) (*t* = 4.05; df = 8; *P* = 0.0155 and *t* = 8.58; df = 8; *P* = 0.0001, respectively; Table 7), which suggests thiamethoxam caused a slight upregulation of expression under drought stress. Lastly, LIP and SAM, a lipoxygenase and SAM dependent carboxyl methyltransferase (respectively), displayed a significant seed treatment × drought stress interaction at 7d (*F* = 37.65; df = 1, 8; *P* = 0.0003 and *F* = 114.87; df = 1, 8; *P* = 0.0048, respectively; Table 7). LIP, a lipoxygenase associated with JA, showed decreased expression (-6.0-fold) under drought stress in thiamethoxam-treated plants compared (*t* = 7.14; df = 8; *P* = 0.0004) to a 1.5-fold upregulation in the untreated control under drought stress (Figure 2). SAM expression followed a similar expression pattern and was significantly repressed (-4.2-fold) in thiamethoxam-treated plants under drought stress (*t* = 4.52; df = 8; *P* = 0.0084) compared to a 1.3-fold upregulation in the untreated controls (Table 8).

## Discussion

It is recognized that the success of the neonicotinoid insecticides is largely due to their broad-spectrum control of arthropod pests in many agronomic cropping systems [2]. However, the potential for these insecticides to influence plant health and provide protection from environmental stress has only recently been suggested [3]. We are only beginning to understand the neonicotinoid stress shield concept at a molecular level, especially the large-scale transcriptional changes. Our study investigated this concept with the neonicotinoid thiamethoxam in order to provide a basis for understanding the molecular underpinnings of this induced stress shield [3].

### Transcriptional response to thiamethoxam seed treatment in absence of stress

This study incorporated a thiamethoxam seed treatment to measure transcriptional changes via RNA-Seq in soybean in the absence of stress. Our findings provide a baseline understanding of the soybean’s response to a thiamethoxam seed treatment. Overall, transcriptional changes induced by the seed treatment were different at each developmental stage with the same genes and pathways not consistently affected by thiamethoxam. This was not unexpected since it is well established that broad-scale transcriptional differences across growth stages are a normal part of plant development, including many changes caused by phytohormones [18–20]. We did not find evidence that thiamethoxam causes consistent transcriptional changes across developmental stages, instead our results suggest that the effects of thiamethoxam on gene expression are unique to each stage.

An aspartic protease (*Glyma11g03500*) was the only gene shared across all three thiamethoxam-treated vegetative stage comparisons (VC-V2, VC-V4, and V2-V4; Figure 1). Aspartic proteases have been shown to be an active part of inducible resistance mechanisms in response to plant pathogens, like *Pseudomonas syringae,* in *A. thaliana* [21]. Its response to thiamethoxam and importance in soybean suggests this gene is an excellent candidate for future studies. We were also interested in genes associated with phytohormones and oxidative enzymes due to their importance in plant defense response [18]. Previously, imidacloprid has been shown to induce genes involved with the salicylic acid pathway [5], an important defense mechanism for environmental stress protection [22]. We found some evidence to support our hypothesis that thiamethoxam will affect plant defenses and oxidation-reduction mechanisms; however, the majority of these genes were downregulated in thiamethoxam-treated plants. It was also revealed that most of these genes were similarly responsive in untreated control plants, indicating that thiamethoxam did not significantly alter the expression of the major defensive pathways of interest.

### Interaction between thiamethoxam seed treatment and drought stress

The stress shield concept was initially investigated by assessing the phenotypic effects of seed treatment and drought stress. Drought stress delayed vegetative development and negatively affected plant biomass and shoot height in both thiamethoxam-treated and untreated soybeans at all three time points (7, 10, and 13d). Previously, thiamethoxam was shown to increase root development, protein content, shoot dry matter and ear dry weight in seed-treated wheat under greenhouse conditions with adequate water [14]. In the present study, a thiamethoxam seed treatment did not show any added health benefits to soybean biomass and shoot height under optimal growing or drought stress conditions.

The effects of the seed treatment and drought stress were only quantified during vegetative stages of soybean development, and therefore long-term impacts were not evaluated in this study. In addition to the negative impacts of stress on physiology, interactions between seed treatment and drought stress were also observed at the molecular level (Table 7). We found five cases to support our hypothesis that thiamethoxam affects gene expression under drought stress. Of particular interest was GmDREB2A;2, which has been documented as inducing the expression of dehydration-responsive elements in *A. thaliana* under drought stress [17]. In our study, the expression of this gene was consistently repressed across time points by thiamethoxam and upregulated in the untreated controls under drought stress (Figure 2). Further studies with other drought-responsive genes are needed to investigate the stress shield concept. If reduced expression similar to that observed for GmDREB2A;2 in the current study is found in additional drought-responsive genes, this may suggest thiamethoxam could limit a plant’s ability to perceive and respond to stress.

It is important to note that plants might respond differently to thiamethoxam than to imidacloprid or clothianidin. As previously discussed, both imidacloprid and clothianidin induced the genes associated with the SA pathway [5]. We failed to identify thiamethoxam-induced genes that were associated with the SA pathway in the presence and absence of drought stress. We did, however, identify genes associated with other phytohormones (ABA, JA, and GA) that were affected by thiamethoxam and warrant further investigation. More research is needed to investigate whether the gene expression patterns observed in this study are also reflected in phytohormone levels in the soybean leaves in response to drought stress. It is also important to consider that other phytohormone signaling pathways may be upregulated with the thiamethoxam seed treatment during alternative abiotic and biotic stresses, such as salt stress or insect herbivory [23, 24].

## Conclusions

This study provides insight into the effect of a thiamethoxam seed treatment on gene expression in soybean. Transcriptional changes observed across vegetative stages in thiamethoxam-treated plants were primarily stage-specific. When drought stress was imposed on the soybean, gene expression was affected by both thiamethoxam and drought stress. Interactive effects between these two treatments were also observed, with genes in thiamethoxam-treated plants showing both induced (THIZ2 and GRP) and repressed (GmDREB2A;2, LIP, and SAM) expression under drought stress relative to untreated plants. In this study, thiamethoxam had a minimal effect on the upregulation of genes associated with phytohormones previously associated with the plant stress shield concept [5]. However, thiamethoxam was not found to enhance plant health in the presence or absence of drought stress, which may in part explain this minimal induction of defense mechanisms.

## Methods

### Experiment I: transcriptional response to thiamethoxam seed treatment

#### Seed treatment and plant material

Soybean seeds (LG Seeds 2699RR [soybean aphid-susceptible]) were treated in the laboratory with Cruiser® 5FS (thiamethoxam, (E, Z)-3-(2-chloro-1,3-thiazol-5-ylmethyl)-5-methyl-1,3,5-oxadiazinan-4-ylidene(nitro)amine) at the labeled rate of 83 mL/l00 kg of seed. Both thiamethoxam-treated and untreated plants were grown in Fafard Growing Media (Mix No. 3B; Conrad Fafard, Awawam, MA). Plant samples for RNA-Seq were grown in plastic nursery pots (15.2 cm diameter × 15.2 cm depth; Reb Plastics Inc., Cleveland, OH) and maintained in a growth chamber (23 ± 2°C, 75 ± 5% RH, 16:8 [L:D] h).

The experiment design was a 2 × 3 factorial arrangement. The main effects were seed treatment (thiamethoxam-treated and untreated control) and vegetative stage (VC, V2, and V4), with three replicates of each treatment arranged in a completely randomized design. Plants from each treatment were harvested at the VC, V2, and V4 stages (unrolled unifoliate leaves, fully developed trifoliate at second and fourth node, respectively; [25]). At the VC stage, plants were trimmed at the base in order to obtain enough plant material, flash frozen in liquid nitrogen, transferred to the laboratory on dry ice, and stored at -80°C until further processing. At the V2 and V4 stages, the top two trifoliates were removed and processed in the same manner.

### Sample preparation and RNA-Seq

Total RNA was extracted from leaf tissues with TRIzol Reagent (Invitrogen Corp, Carlsbad, CA) according to the manufacturer’s protocol and purified using Qiagen® RNeasy Mini Kit (Qiagen, Valencia, CA). Initially, two V2 tissue samples, one each of thiamethoxam-treated and untreated control plant, were submitted for sequencing to compare differential gene expression. Sample libraries were then submitted to the Center for Biotechnology at the University of Nebraska-Lincoln for RNA-Seq on the Illumina Genome Analyzer IIx. Sample preparation and analysis followed the manufacturer’s protocol (Illumina Inc., San Diego, CA). This process used two 36-cycle sequencing kits with each sample analyzed on a single lane to generate an average of 28 million single-end (75 base pair [bp]) reads. The remaining 16 samples were submitted to the University of Nebraska Medical Center Genomics Core facility (Omaha, NE) for sequencing on the Illumina HiSeq 2000. This included three replicates for thiamethoxam-treated and three replicates for untreated VC and V4 samples. Two replicates for thiamethoxam-treated and two replicates for untreated V2 samples were also submitted. Four samples were analyzed on a single lane. The HiSeq generated an average of 29 million single-end (100 bp) reads. Although differences between the two Illumina technologies exist, the sequencing depth was similar and therefore data were combined for subsequent mapping and analysis (three replicates total for V2).

### Gene expression analysis

The obtained sequence reads were trimmed for adapter sequences and verified for quality. Gene expression was estimated by mapping reads to the soybean genome (*G. max* 109, phytozome.net) using Bowtie 2 (version 2.0.0-beta5; [26]). Protein homologues were identified using Blast2GO, which searched contigs against the GenBank non-redundant database using BLASTp algorithms, and implementing Gene Ontology (GO) annotation. Splice variants were removed from the fully annotated file in order to reduce redundancy of GO IDs.

Differential gene expression was evaluated with pairwise comparisons using DESeq [27] in the *R* statistical environment (version 3.0.2; [28]), where the *P*-value was adjusted for multiple testing by controlling the false discovery rate (FDR) at 0.05% [29]. These genes were selected for GO enrichment analyses included only those with a fold change ≥ 2.0 and were categorized as either up- or downregulated. All enrichment analyses were performed in Blast2GO using Fisher’s Exact Test (FDR ≤ 0.10) for genes identified as DE between 1) thiamethoxam-treated and untreated control plants and 2) vegetative stages within experimental groups.

### qRT-PCR validation of RNA-Seq results

Four target genes were selected based on large upregulation (≈10 – 200-fold changes) and consistent differential expression in most of the vegetative stage comparisons or possible roles in stress response, including ones previously identified in soybean leaf tissues [15]. A gibberellin regulated protein (GRP), thiamine biosynthetic enzyme (THIZ1), WRKY51 DNA-binding protein 51 (WKRY51) and cartenoid oxygenase (CAR) were selected and a cyclophilin type peptidyl-prolyl cis-trans isomerase (CYP2) was used as a reference gene [30, 31]. Primer3 software was used to design primers (Additional file 4: Table S4). In accordance with the manufacturer’s protocol, cDNA was prepared from 2 μg of total RNA (ThermoScript RT-PCR System; Invitrogen). Primer efficiencies were calculated from the linear regression of log transformed expression values obtained from a series of four, 10-fold dilutions of cDNA.

Quantitative RT-PCR was performed with TaqMan® assay in a 7500 Fast Real-Time PCR System according to manufacturer’s instructions (Applied Biosystems, Foster City, CA). The plate setup included inter-run calibrations in order to estimate and correct for plate to plate variation, and relative expression level for each gene was measured following previously developed methods [32].

The CNRQs were assessed for normality using a Shapiro-Wilk test [33]. When appropriate, a log transformation was performed on non-normalized data for statistical analyses and reconverted to the original scale for summarization in the tables. Differences in gene expression (CNRQs) among the vegetative stages (VC-V2, VC-V4, and V2-V4) within each seed treatment were analyzed using ANOVA, implemented in SAS PROC GLIMMIX (SAS Institute 2006, Cary, NC). When appropriate, means were separated using Fisher’s least significant difference (LSD) test (α = 0.05) with a Tukey adjustment for multiple comparisons. Separately, the transcript levels from the RNA-Seq data for each of the four target genes were converted to reads per kilobase of exon model per million mapped reads (RPKM; [34]). A correlation between thiamethoxam-treated and untreated control treatment means at each vegetative stage of the RPKM and CNRQs (from RNA-Seq and qRT-PCR data, respectively) was calculated globally across all genes.

### Experiment II: impact of drought stress on gene expression

#### Seed treatment, plant material and experimental design

Soybean seeds were treated with the same rate of Cruiser® 5FS as in Experiment I (83 ml/l00 kg of seed). Plants were grown in Fafard Growing Media (Mix No. 3B; Conrad Fafard, Awawam, MA) in plastic nursery pots (10.1 cm diameter × 12.7 cm depth; Zarn Inc., Reidsville, NC) and maintained in greenhouse conditions (25 ± 5°C, 75 ± 5% RH, 16:8 [L:D] h). The experimental design was a 2 × 2 × 3 factorial arrangement that included seed treatment (thiamethoxam-treated and untreated control), stress level (drought stress and no stress) and three sampling time points (7, 10, 13d), each with 11 total replicate plants (eight used for the physiological measurements of plant health and three for gene expression analysis [qRT-PCR]). All plants were placed in a completely randomized design and randomly moved daily in order to minimize effects of variation due to placement on the greenhouse bench.

### Application of drought stress

All seeds (thiamethoxam-treated and untreated) were planted in pots (132 total) with 200 g of soil and placed in plastic trays with ≈ 7.6 cm of water. This provided a constant water source and allowed for complete saturation of the soil over the growth period from germination to the VC stage. The average weight of the saturated soil was 550 g prior to the application of drought stress. At the VC stage drought stress was applied to half of the plants (66 total: 33 treated, 33 untreated) by removing them from the plastic trays and no longer providing water for the duration of their development (13 additional days). Drought stress was applied by allowing the soil to gradually dry over the course of the experiment.

Plants from each experimental treatment group were harvested seven, ten and thirteen days after stress was imposed at the VC stage (7, 10, and 13d). We quantified the decrease in soil moisture for stressed plants by randomly selecting 20 pots and measuring soil weight daily. At the first sampling point (7d), the soil weight of moisture stressed plants weighed 210 ± 10 g less than unstressed plants that were provided a continuous source of water. For the remaining six days of the study, the soil drought in the stressed pots decreased ≈ 15 g per day. The observed decrease in soil weight demonstrates that drought stress was gradually applied over the duration of the study relative to unstressed plants which maintained a constant soil weight of 550 g.

### Quantification and analysis of the effects of drought stress on soybean physiology

Developmental rate, plant biomass (i.e., the difference between wet and dry weight), and shoot height were our three selected parameters for measuring the effect of drought stress. Stress level was measured as the reduction in these physiological parameters relative to unstressed plants. Plants were destructively sampled at each time point (7, 10, and 13d) with eight replicates per treatment. Measurements of plant health were analyzed at each time point separately because the level of drought stress was not applied at a constant level and predicted to increase as the soil dried. This resulted in heterogeneity of variance across time points. Plant biomass and shoot height were analyzed at each separate time point using an ANOVA (SAS PROC GLIMMIX) with the following factors: seed treatment (thiamethoxam-treated and untreated), stress (drought stress and no stress) and the interaction between seed treatment and stress (seed treatment × drought stress). In cases where a significant two-way interaction was found, means were separated using Fisher’s LSD test (α = 0.05) with a Tukey correction for multiple testing. The plants were also observed regularly for phenotypic signs of stress, including leaf curling and delayed vegetative development.

### Selection of drought stress and thiamethoxam responsive genes

For gene expression analysis the first and second trifoliates were removed from each plant and handled in the same manner as described previously for the RNA-Seq study. Ten target genes were selected based on possible response to thiamethoxam or drought stress, including a lipoxygenase (LIP), SAM dependent carboxyl methyltransferase (SAM), aspartic protease (AP), apetala 2 (GmDREB2A;2), cytochrome P450 (P450), two thiamine biosynthetic enzymes (THIZ1 and THIZ2), gibberellin regulated protein (GRP), WRKY51 DNA-binding protein 51 (WKRY51) and cartenoid oxygenase (CAR). LIP and SAM are jasmonic and salicylic acid-associated genes, respectively [15] and GmDREB2A;2 was previously documented to be drought stress responsive in soybean [17].

### Expression analysis of drought stress and thiamethoxam responsive genes

Gene expression was calculated using the CNRQs, as previously described for RNA-Seq validation using qRT-PCR. The expression of each of the 10 target genes was analyzed separately at each time point (7, 10, and 13d) using an ANOVA (SAS PROC GLIMMIX). Means were separated using Fisher’s LSD test (α = 0.05) when appropriate. The main effects of the model were seed treatment (thiamethoxam-treated and untreated control), stress (drought stress and no stress) and the seed treatment × stress interaction.

### Accession numbers

The accession numbers of the cDNA sequences are as follows: GmDREB2A;2 (JX440387), AP (XM_006591467), CAR (XR_418624), CYP2 (NM_001248294), GRP (XM_003525870), LIP (XM_003555992), P450 (XM_003554975), SAM (XM_003548827), THIZ1 (XM_003536502), THIZ2 (XM_003555980) and WRKY61 (XM_003526787).

## Electronic supplementary material

Additional file 1: Table S1: Enrichment analysis for GO Terms (FDR < 0.10) of unique DE genes, which were upregulated in the untreated VC-V2, VC-V4, and V2-V2 comparisons. The 20 most abundant processes are presented. (DOC 72 KB)

Additional file 2: Table S2: Enrichment analysis for GO Terms (FDR < 0.10) of unique DE genes, which were downregulated in the untreated VC-V2, VC-V4, and V2-V2 comparisons. The 20 most abundant processes are presented. (DOC 70 KB)

Additional file 3: Table S3: Validation of RNA-Seq by qRT-PCR for thiamethoxam-treated VC-V2, VC-V4, and V2-V4 comparisons. Validation of RNA-Seq by qRT-PCR for untreated VC-V2, VC-V4, and V2-V4 comparisons. (DOC 44 KB)

Additional file 4: Table S4: Primer pair design RNA-Seq validation and gene expression analysis using qRT-PCR. (DOC 41 KB)
