# Supplementary material for: Transcriptional response of soybean to thiamethoxam seed treatment in the presence and absence of drought stress
Source: BMC Genomics. 2014 Dec 3;15(1):1055. doi: 10.1186/1471-2164-15-1055 (PMC4265413; doi:10.1186/1471-2164-15-1055)
Supplement: Supplementary file 1 — Additional file 1: Table S1: Enrichment analysis for GO Terms (FDR < 0.10) of unique DE genes, which were upregulated in the untreated VC-V2, VC-V4, and V2-V2 comparisons. The 20 most abundant processes are presented. (DOC 72 KB) [file 12864_2014_6726_MOESM1_ESM.doc]

**Additional file 1: Table S1** Enrichment analysis for GO Terms (FDR < 0.10) of unique DE genes, which were upregulated in the untreated VC-V2, VC-V4, and V2-V2 comparisons. The 20 most abundant processes are presented.

a. Untreated VC-V2

| **GO-ID** | **GO Term** | **FDR** | **# Sequences** |
| --- | --- | --- | --- |
| GO:0044699 | single-organism process | 6.93E-02 | 302 |
| GO:0044710 | single-organism metabolic process | 5.79E-02 | 199 |
| GO:0044711 | single-organism biosynthetic process | 1.67E-02 | 53 |
| GO:0016757 | transferase activity, transferring glycosyl groups | 3.52E-02 | 48 |
| GO:0044085 | cellular component biogenesis | 9.31E-02 | 42 |
| GO:0048037 | cofactor binding | 9.91E-02 | 41 |
| GO:0071554 | cell wall organization or biogenesis | 2.76E-06 | 40 |
| GO:0010154 | fruit development | 6.49E-02 | 40 |
| GO:0005576 | extracellular region | 9.34E-02 | 35 |
| GO:0009793 | embryo development ending in seed dormancy | 9.34E-02 | 31 |
| GO:0071669 | plant-type cell wall organization or biogenesis | 1.06E-03 | 23 |
| GO:0005976 | polysaccharide metabolic process | 9.34E-02 | 23 |
| GO:0042546 | cell wall biogenesis | 1.53E-05 | 22 |
| GO:0005507 | copper ion binding | 6.61E-02 | 22 |
| GO:0034637 | cellular carbohydrate biosynthetic process | 4.29E-02 | 21 |
| GO:0044264 | cellular polysaccharide metabolic process | 9.19E-02 | 21 |
| GO:0071555 | cell wall organization | 4.89E-02 | 19 |
| GO:0045229 | external encapsulating structure organization | 9.31E-02 | 19 |
| GO:0033692 | cellular polysaccharide biosynthetic process | 4.80E-02 | 18 |
| GO:0000271 | polysaccharide biosynthetic process | 5.27E-02 | 18 |

b. Untreated VC-V4

| **GO-ID** | **GO Term** | **FDR** | **# Sequences** |
| --- | --- | --- | --- |
| GO:0044464 | cell part | 8.36E-05 | 1958 |
| GO:0005623 | cell | 8.36E-05 | 1958 |
| GO:0005488 | binding | 1.13E-03 | 1722 |
| GO:0005622 | intracellular | 2.25E-03 | 1694 |
| GO:0009987 | cellular process | 1.10E-04 | 1660 |
| GO:0044424 | intracellular part | 2.80E-03 | 1649 |
| GO:0043229 | intracellular organelle | 1.18E-05 | 1554 |
| GO:0043226 | organelle | 1.38E-05 | 1554 |
| GO:0043231 | intracellular membrane-bounded organelle | 1.19E-02 | 1445 |
| GO:0043227 | membrane-bounded organelle | 1.27E-02 | 1445 |
| GO:0044237 | cellular metabolic process | 2.95E-02 | 1240 |
| GO:0044238 | primary metabolic process | 4.35E-02 | 1237 |
| GO:0044699 | single-organism process | 2.49E-14 | 1077 |
| GO:0097159 | organic cyclic compound binding | 1.08E-08 | 1074 |
| GO:1901363 | heterocyclic compound binding | 8.87E-09 | 1073 |
| GO:0043170 | macromolecule metabolic process | 4.11E-06 | 983 |
| GO:0044260 | cellular macromolecule metabolic process | 7.62E-08 | 923 |
| GO:0044763 | single-organism cellular process | 1.63E-16 | 877 |
| GO:0006807 | nitrogen compound metabolic process | 3.14E-12 | 726 |
| GO:1901360 | organic cyclic compound metabolic process | 1.32E-16 | 711 |

c. Untreated V2-V4

| **GO-ID** | **GO Term** | **FDR** | **# Sequences** |
| --- | --- | --- | --- |
| GO:0005488 | binding | 1.62E-02 | 1366 |
| GO:0044699 | single-organism process | 8.60E-16 | 890 |
| GO:0097159 | organic cyclic compound binding | 1.59E-06 | 853 |
| GO:1901363 | heterocyclic compound binding | 1.44E-06 | 852 |
| GO:0043170 | macromolecule metabolic process | 2.58E-04 | 779 |
| GO:0044763 | single-organism cellular process | 1.74E-16 | 722 |
| GO:0044260 | cellular macromolecule metabolic process | 1.89E-04 | 719 |
| GO:0006807 | nitrogen compound metabolic process | 7.52E-09 | 574 |
| GO:1901360 | organic cyclic compound metabolic process | 1.37E-12 | 565 |
| GO:0006725 | cellular aromatic compound metabolic process | 8.62E-13 | 552 |
| GO:0046483 | heterocycle metabolic process | 2.69E-13 | 546 |
| GO:0034641 | cellular nitrogen compound metabolic process | 1.55E-12 | 546 |
| GO:0006139 | nucleobase-containing compound metabolic process | 5.65E-16 | 526 |
| GO:0065007 | biological regulation | 4.80E-06 | 503 |
| GO:0005634 | nucleus | 5.70E-23 | 501 |
| GO:0050789 | regulation of biological process | 8.19E-08 | 490 |
| GO:0016787 | hydrolase activity | 7.41E-03 | 464 |
| GO:0000166 | nucleotide binding | 7.04E-02 | 461 |
| GO:1901265 | nucleoside phosphate binding | 7.04E-02 | 461 |
| GO:0003676 | nucleic acid binding | 1.83E-08 | 456 |
