# Supplementary material for: Transcriptional response of soybean to thiamethoxam seed treatment in the presence and absence of drought stress
Source: BMC Genomics. 2014 Dec 3;15(1):1055. doi: 10.1186/1471-2164-15-1055 (PMC4265413; doi:10.1186/1471-2164-15-1055)
Supplement: Supplementary file 2 — Additional file 2: Table S2: Enrichment analysis for GO Terms (FDR < 0.10) of unique DE genes, which were downregulated in the untreated VC-V2, VC-V4, and V2-V2 comparisons. The 20 most abundant processes are presented. (DOC 70 KB) [file 12864_2014_6726_MOESM2_ESM.doc]

**Additional file 2: Table S2** Enrichment analysis for GO Terms (FDR < 0.10) of unique DE genes, which were downregulated in the untreated VC-V2, VC-V4, and V2-V2 comparisons. The 20 most abundant processes are presented.

a. Untreated VC-V2

| **GO-ID** | **GO Term** | **FDR** | **# Sequences** |
| --- | --- | --- | --- |
| GO:0016491 | oxidoreductase activity | 1.33E-03 | 95 |
| GO:0055114 | oxidation-reduction process | 6.37E-02 | 75 |
| GO:0046906 | tetrapyrrole binding | 7.79E-04 | 28 |
| GO:0020037 | heme binding | 7.79E-04 | 27 |
| GO:0009055 | electron carrier activity | 5.09E-02 | 26 |
| GO:0004497 | monooxygenase activity | 4.07E-02 | 20 |
| GO:0010243 | response to organonitrogen compound | 5.09E-02 | 12 |
| GO:0004857 | enzyme inhibitor activity | 3.38E-02 | 11 |
| GO:0010200 | response to chitin | 4.07E-02 | 11 |
| GO:0031408 | oxylipin biosynthetic process | 1.11E-03 | 8 |
| GO:0031407 | oxylipin metabolic process | 1.11E-03 | 8 |
| GO:0070330 | aromatase activity | 8.12E-03 | 7 |
| GO:0016712 | oxidoreductase activity, acting on paired donors, incorporation/reduction of molecular oxygen, reduced flavin/flavoprotein, incorporation of one atom of oxygen | 8.12E-03 | 7 |
| GO:0008794 | arsenate reductase (glutaredoxin) activity | 1.33E-03 | 6 |
| GO:0030611 | arsenate reductase activity | 2.02E-03 | 6 |
| GO:0030613 | oxidoreductase activity, acting on phosphorus or arsenic in donors | 1.33E-03 | 6 |
| GO:0030614 | oxidoreductase activity, acting on phosphorus or arsenic in donors, disulfide as acceptor | 1.33E-03 | 6 |
| GO:0033773 | isoflavone 2'-hydroxylase activity | 9.07E-02 | 3 |

b. Untreated VC-V4

| **GO-ID** | **GO Term** | **FDR** | **# Sequences** |
| --- | --- | --- | --- |
| GO:0008152 | metabolic process | 6.68E-02 | 993 |
| GO:0003824 | catalytic activity | 8.17E-05 | 951 |
| GO:0005737 | cytoplasm | 9.40E-03 | 800 |
| GO:0044444 | cytoplasmic part | 7.50E-02 | 729 |
| GO:0016020 | membrane | 2.72E-04 | 579 |
| GO:0043167 | ion binding | 1.46E-04 | 561 |
| GO:0044710 | single-organism metabolic process | 1.96E-13 | 455 |
| GO:0050896 | response to stimulus | 4.91E-05 | 445 |
| GO:0009536 | plastid | 2.05E-11 | 421 |
| GO:0009507 | chloroplast | 2.06E-17 | 349 |
| GO:0016787 | hydrolase activity | 4.49E-02 | 343 |
| GO:0043168 | anion binding | 9.16E-03 | 320 |
| GO:0044446 | intracellular organelle part | 7.08E-03 | 316 |
| GO:0044422 | organelle part | 7.97E-03 | 316 |
| GO:0043169 | cation binding | 3.21E-02 | 289 |
| GO:0051179 | localization | 1.80E-06 | 277 |
| GO:0032553 | ribonucleotide binding | 1.79E-02 | 272 |
| GO:0051234 | establishment of localization | 3.17E-06 | 271 |
| GO:0006810 | transport | 3.55E-06 | 269 |
| GO:0046872 | metal ion binding | 5.97E-02 | 269 |

c. Untreated V2-V4

| **GO-ID** | **GO Term** | **FDR** | **# Sequences** |
| --- | --- | --- | --- |
| GO:0008152 | metabolic process | 9.80E-04 | 859 |
| GO:0003824 | catalytic activity | 3.12E-11 | 850 |
| GO:0016020 | membrane | 5.32E-05 | 496 |
| GO:0044699 | single-organism process | 8.22E-02 | 481 |
| GO:0043167 | ion binding | 1.21E-04 | 475 |
| GO:0044710 | single-organism metabolic process | 9.79E-18 | 409 |
| GO:0050896 | response to stimulus | 1.90E-02 | 352 |
| GO:0009536 | plastid | 1.60E-08 | 347 |
| GO:0009507 | chloroplast | 4.19E-18 | 307 |
| GO:0016787 | hydrolase activity | 2.44E-03 | 304 |
| GO:0043169 | cation binding | 4.58E-05 | 270 |
| GO:0051179 | localization | 6.95E-11 | 260 |
| GO:0006810 | transport | 1.22E-11 | 258 |
| GO:0051234 | establishment of localization | 2.66E-11 | 258 |
| GO:0016491 | oxidoreductase activity | 1.08E-12 | 256 |
| GO:0046872 | metal ion binding | 1.40E-03 | 244 |
| GO:0044425 | membrane part | 2.85E-07 | 238 |
| GO:0044765 | single-organism transport | 3.05E-18 | 231 |
| GO:0055114 | oxidation-reduction process | 3.88E-13 | 227 |
| GO:0031224 | intrinsic to membrane | 2.58E-09 | 210 |
