# Supplementary material for: Transcriptional response of soybean to thiamethoxam seed treatment in the presence and absence of drought stress
Source: BMC Genomics. 2014 Dec 3;15(1):1055. doi: 10.1186/1471-2164-15-1055 (PMC4265413; doi:10.1186/1471-2164-15-1055)
Supplement: Supplementary file 3 — Additional file 3: Table S3: Validation of RNA-Seq by qRT-PCR for thiamethoxam-treated VC-V2, VC-V4, and V2-V4 comparisons. Validation of RNA-Seq by qRT-PCR for untreated VC-V2, VC-V4, and V2-V4 comparisons. (DOC 44 KB) [file 12864_2014_6726_MOESM3_ESM.doc]

**Additional file 3: Table S3** Validation of RNA-Seq by qRT-PCR for thiamethoxam-treated VC-V2, VC-V4, and V2-V4 comparisons.

|  | **Thiamethoxam VC-V2** | | | | **Thiamethoxam VC-V4** | | | | **Thiamethoxam V2-V4** | | | |
| --- | --- | --- | --- | --- | --- | --- | --- | --- | --- | --- | --- | --- |
|  | **qRT-PCR** | | **RNA-Seq** | | **qRT-PCR** | | **RNA-Seq** | | **qRT-PCR** | | **RNA-Seq** | |
| **Gene** | **FC^a^** | ***p* adj^b^** | **FC** | ***p* adj** | **FC** | ***p* adj** | **FC** | ***p* adj** | **FC** | ***p* adj** | **FC** | ***p* adj** |
| GRP | 141.49 | <0.0001 | 195.49 | <0.0001 | 273.05 | <0.0001 | 130.75 | 0.0012 | 1.93 | 1.0000 | -1.52 | 0.6550 |
| THIZ1 | 3.07 | 0.9906 | 10.75 | <0.0001 | 50.22 | 0.0147 | 75.07 | <0.0001 | 16.33 | 0.8538 | 6.89 | 0.0128 |
| WRKY51 | 14.40 | 0.478 | 46.33 | <0.0001 | 12.70 | 0.0392 | 11.49 | 0.0036 | -1.13 | 1.0000 | -4.11 | 0.0975 |
| CAR | 1.08 | 1.000 | 3.98 | <0.0001 | 4.08 | 0.9749 | 1.85 | 0.3121 | 3.79 | 0.9971 | -2.21 | 0.0411 |

^a^ FC = Fold Change.

^b^ *p*-value adjusted for a false discovery rate (FDR) of 0.10.

**Additional file 3: Table S3** Validation of RNA-Seq by qRT-PCR for untreated VC-V2, VC-V4, and V2-V4 comparisons.

|  | **Untreated VC-V2** | | | | **Untreated VC-V4** | | | | **Untreated V2-V4** | | | |
| --- | --- | --- | --- | --- | --- | --- | --- | --- | --- | --- | --- | --- |
|  | **qRT-PCR** | | **RNA-Seq** | | **qRT-PCR** | | **RNA-Seq** | | **qRT-PCR** | | **RNA-Seq** | |
| **Gene** | **FC^a^** | ***p* adj^b^** | **FC** | ***p* adj** | **FC** | ***p* adj** | **FC** | ***p* adj** | **FC** | ***p* adj** | **FC** | ***p* adj** |
| GRP | 58.86 | <0.0001 | 98.04 | <0.0001 | 216.85 | <0.0001 | 57.24 | 0.0372 | 3.68 | 1.0000 | -1.72 | 0.4557 |
| THIZ1 | 3.83 | 0.7637 | 10.13 | <0.0001 | -1.49 | 1.0000 | 17.72 | 0.0004 | -5.71 | 0.4030 | 1.74 | 0.4443 |
| WRKY51 | 7.01 | 0.0980 | 17.78 | <0.0001 | 18.60 | 0.0059 | 7.27 | <0.0001 | 2.66 | 0.9995 | -2.46 | 0.0002 |
| CAR | 1.72 | 1.0000 | 3.05 | <0.0001 | 7.22 | 0.4436 | 1.96 | 0.0385 | 4.21 | 0.8966 | -1.55 | 0.1065 |

^a^ FC = Fold Change.

^b^ *p*-value adjusted for a false discovery rate (FDR) of 0.10.
