# Supplementary material for: Transcriptional response of soybean to thiamethoxam seed treatment in the presence and absence of drought stress
Source: BMC Genomics. 2014 Dec 3;15(1):1055. doi: 10.1186/1471-2164-15-1055 (PMC4265413; doi:10.1186/1471-2164-15-1055)
Supplement: Supplementary file 4 — Additional file 4: Table S4: Primer pair design RNA-Seq validation and gene expression analysis using qRT-PCR. (DOC 41 KB) [file 12864_2014_6726_MOESM4_ESM.doc]

**Additional file 4: Table S4** Primer pair design RNA-Seq validation and gene expression analysis using qRT-PCR.

| **Target Gene  *ID*** | **Primer**  **Type** | **Primer sequence 5’-3’** | **Amplicon (bp)** | **Primer Efficiency**  **(%)** | **R^2^** |
| --- | --- | --- | --- | --- | --- |
| GmDREB2A;2  *Glyma14g06080* | F  R | AGGGAGCCCAATAGAGGAAG  CTGCCGAAGAACAAGAGGAC | 202 | 205.8 | 0.99 |
|  |  |  |  |  |  |
| AP  *Glyma11g03500* | F  R | TCACCGGAATCTCTGTAGGG  TTTTTCCTCCACCTCACTCG | 203 | 190.4 | 0.99 |
|  |  |  |  |  |  |
| CAR  *Glyma15g40070* | F  R | CGACGAGATTGTTGTGATCG  CTTTCGTCCGAGCTTGTTTC | 202 | 131.7 | 1.32 |
|  |  |  |  |  |  |
| CYP2  *Glyma12g02790* | F  R | CCCCTCCACTACAAAGGCTCG  CGGGACCAGTGTGCTTCTTCA | 154 | 193.3 | 1.00 |
|  |  |  |  |  |  |
| Cyto P450  *Glyma19g09290* | F  R | CGTGGAATCGATTAGCAGGT  CTTCGCCCCATATTTCCTCT | 197 | 227.7 | 2.28 |
|  |  |  |  |  |  |
| GRP *Glyma06g04740* | F  R | GATGCTTGAGGTTTTGCGGG  GCATTTGTCCTTGCCCTTGG | 125 | 110.7 | 1.10 |
| LIP  *Glyma20g28290* | F  R | GCCAAGTGGACCTTTAACGA  GGAGACGGCGGATAATAACA | 203 | 189.0 | 0.99 |
|  |  |  |  |  |  |
| SAM  *Glyma16g24740* | F  R | GTTGCTCTTCTGGGCAACAT  CATGGACCAATCCCACTTTC | 208 | 204.2 | 0.99 |
|  |  |  |  |  |  |
| THIZ1 *Glyma10g39740* | F  R | CATGTCCCTAACCCAACCCC  GGACTGCTCGATTATGGCGA | 219 | 105.0 | 1.05 |
|  |  |  |  |  |  |
| THIZ2 *Glyma20g27990* | F  R | ATGCTGCTGAGGACGCTATT  CAACTCCACACGTTCCATCA | 20 | 191.8 | 0.99 |
|  |  |  |  |  |  |
| WKRY51 *Glyma06g15220* | F  R | CCCAACCCACGGAACTACTA  GAGTTTGCAGAAGGGTGGAG | 203 | 112.7 | 1.13 |
